# Lipoprotein (a), C-reactive protein and some metabolic cardiovascular risk factors in type 2 DM

**DOI:** 10.1186/1758-5996-2-51

**Published:** 2010-07-27

**Authors:** Anthonia O Ogbera, Alfred O Azenabor

**Affiliations:** 1 Department of Medicine, Lagos State University Teaching Hospital, Ikeja, Lagos, Nigeria; 2 Department of Medicine, General Hospital Gbagada, Lagos, Nigeria; 3 Department of Surgery and Chemical Pathology, Lagos University Teaching Hospital, Idi-araba, Lagos, Nigeria

## Abstract

**Background:**

Lipoprotein (a) (LP (a) is an independent cardiovascular risk factor that is not widely studied in people of sub-Saharan African origin. The aim of this report is to determine the frequency of occurrence of elevated Lp (a) and possible relationship with total cholesterol (TCHOL), high density lipoprotein cholesterol (HDL-C), low density lipoprotein cholesterol (LDL-C), triglycerides (TG), C reactive protein (CRP) and serum uric acid (SUA).

**Methods:**

This is a cross sectional study carried out in 200 Nigerian patients with type 2 DM and 100 sex and age matched healthy Controls aged between 32-86 years. We determined the frequency of occurrence of elevated Lp (a) levels in the study subjects and compared clinical and biochemical variables between type 2 diabetic patients and non-diabetic patients. Clinical and biochemical parameters were also compared between subjects with type 2 DM who had elevated LP (a) and normal LP (a) levels. Long term glycaemic control using glycosylated haemoglobin was determined and compared in the study subjects. Test statistics used include chi square, correlation coefficient analysis and Student's t test.

**Results:**

The mean Lp(a) concentration differed significantly between type 2 diabetic patients and the Control subjects (18.7 (5.8) mg/dl vs 23 (6.8) mg/dl, 0.00001). Similarly, the prevalence of high LP (a) levels in type 2 DM patients was significantly higher than that of the Control subjects (12.5% vs 4%, p-0.019). The mean levels of the lipid profile parameters (TCHOL, LDL-C, TG, LDL/HDL) and CRP were significantly higher in DM patients than in the Control subjects. The mean LP (a) levels were comparable in both sexes and in DM subjects with and without hypertension. TG was the only parameter that differed significantly between subjects with elevated Lp (a) levels and those with normal Lp (a) levels. There was a significant positive correlation (r) between Lp(a) levels and TG, LDL-C. TCHOL, LDL/HDL and uric acid. No association was found between Lp(a) and clinical parameters such as age and anthropometric indices.

**Conclusion:**

We have showed that Lp (a), CRP and other CVS risk factors cluster more in patients with DM than non DM patients. Serum Lp (a) levels are not associated with anthropometric and glycaemic indices.

## Background

Diabetes mellitus is a chronic metabolic disorder that is often associated with unacceptably high disease burden especially in developing countries [1] and cardiovascular (CVS) complications of DM are highly contributory to this scenario. Well studied and documented CVS risk factors in DM include components of the metabolic syndrome namely reduced high density lipoprotein cholesterol and reduced triglyceride levels, central obesity and hypertension [2]. Other CVS risk factors that have not been widely studied in African populations include elevated C-reactive protein, hyperuricaemia and high lipoprotein a levels. Lipoprotein(a) [Lp(a)] consists of an LDL-like particle and the specific apolipoprotein(a) [apo(a)], which is covalently bound to the apoB of the LDL like particle [3]. Elevated levels of Lipoprotein a (Lp (a) ) are found to be independent risk factors for coronary heart disease. The structure of Lp(a) resembles LDL and its atherogenic properties can be explained by its binding to glycosaminoglycans and inhibition of fibrinolysis [4]. The atherogenic properties of Lp(a) are expressed over 30 mg/dL serum concentration [4]. Some reports on serum Lp (a) levels in subjects with type 2 DM show that Lp (a) levels are higher in this group of patients compared with non diabetic healthy controls [5,6].

Elevated C reactive protein (CRP) levels are reported to reflect not only the inflammatory status in type 2 DM but also are associated with other indicators of diabetes-related cardiovascular risk[7]. Elevated serum uric acid (SUA) levels have been associated not only with the components of the Mets [8] but also found to be predictors of cardiovascular diseases in non diabetic patients and those with type 2 diabetes [9,10].

The aim of this report is to determine the frequency and pattern of occurrence of Lp (a) levels in subjects with type 2 DM. Other objectives include comparing the biochemical and clinical parameters of the subjects with type 2 DM who had elevated Lp {a) levels and those without elevated Lp (a) levels.We also sought to determine the prevalence of elevated CRP and SUA levels in subjects with type 2 DM.

## Methods

This study was carried out at the Diabetes Centre of the Lagos State University Teaching Hospital, Ikeja, Lagos State, Nigeria for a period of three months. A total of two hundred patients with type 2 DM and a hundred healthy age and sex matched individuals were recruited as the Control group. Inclusion criteria for subjects with type 2 DM included patients who were treatment naïve for dyslipidaemia. Exclusion criteria for the subjects with type 2 DM included acutely ill patients requiring hospitalization, those with renal failure or already on dialysis.

The study subjects underwent clinical examination that included anthropometric measurements. The anthropometric measurements comprised of waist circumference, height and body weight, and the body mass index (BMI) was calculated as weight/height^2 ^(kg/m^2^). Waist circumference was determined by applying a tape measure to the midpoint between the inferior margin of the last rib and the crest of the ilium.

Biochemical analyses-The DM free status of the controls was ascertained by having them subjected to glycosylated hemoglobin test. They were considered to be non diabetic if their glycosylated haemoglobin levels were less than 6.5%.

The Controls and the subjects with type 2 DM all had some biochemical tests done and these included glycosylated haemoglobin (HbA1c), fasting lipid profile and blood glucose, uric acid and CRP. Lipids, blood glucose and uric acid were analysed spectrophotometrically. The name and model of the spectrophotometer used are SSRFI and BSA 3000.

Lipid: Total cholesterol was determined using a modified method of Liebermann-Burchard [11], HDL-cholesterol by precipitation method [12] and TG was estimated using a kit employing enzymatic hydrolysis of TG with lipases [13]. The Friedwald's formula [14] LDL = (TCHOL - HDL-C) - TG/5 was used to determine LDL-C when the values of TG were less than 400 mg% and LDL-C/HDL-C ratios were calculated. Plasma glucose was measured using the glucose oxidase method[15] and uric acid was measured on a standard autoanalyzer.

Glycosylated haemoglobin level was determined as a point of care test using capillary samples with the Biorad equipment.

CRP and Lp(a) levels were determined using immuno turbidimetric methods.

The intra-assay CVs for SUA, Lp (a) and CRP were 1.66%, 1.68% and .1.52% respectively and the inter-assay CVs were 1.81%, 1.14%, 1.84% and 1.72% respectively.

### Working diagnosis

Elevated Lp (a) levels refer to serum levels above 30 mg/dl [4]

Elevated CRP levels refer to levels above 3 mg%[16]. Hyperuricaemia or elevated SUA levels refer to serum urate levels of > 6 mg/dl in women and > 7 mg/dl in men [17].

### Statistical Analysis

Data were analyzed using SPSS version 17. Student's test was used to compute the mean levels of continuous variables and also to make comparison of these continuous variables between different groups. Pearson correlation coefficient determination was done to evaluate the degree of association between Lp(a ) and clinical and biochemical parameters. Chi square analysis was used to compare proportions. Quantitative data are expressed as mean and standard deviation (SD). P values of < 0.05 were considered to be statistically significant.

## Results

The mean age and standard deviation (SD) of the patients with type 2 DM was 57.7 (10.8) years and the female: male ratio was 134:66. The age range was 32-86 years. The mean and (SD) BMI and waist circumference of the subjects with type 2 DM was 29.7 (7.5) and 93 (12,4) respectively. The mean age (SD) of the Controls was 56.1 (11.2) years and this was comparable to that of the subjects with type 2 DM (p-0.8). A total number of 103 of the subjects with type 2 DM had a history of hypertension thus making up 52% of the population.

### A summary of some clinical and biochemical parameters in subjects with type 2 DM

The mean levels of some clinical and biochemical parameters of the subjects with type 2DM are shown in table 1.

**Table 1:** Clinical and biochemical parameters of subjects with type 2 DM

Variable	Mean(SD)	Range
BMI(Kg/m2)	28.6(5.9)	15-57

WC(cm)	93.1(12.4)	59-140

*DDM (years)	6(5.9)	0.1-9

HbA1c(%)	6.3(2.4)	4-14

FBS(mg%)	163(70)	43-301

*duration of DM

**Figure 1:**
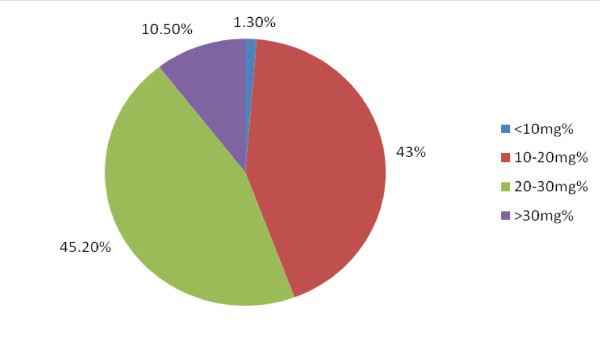
**Distribution of Lp (a) in the study subjects**.

**Figure 2:**
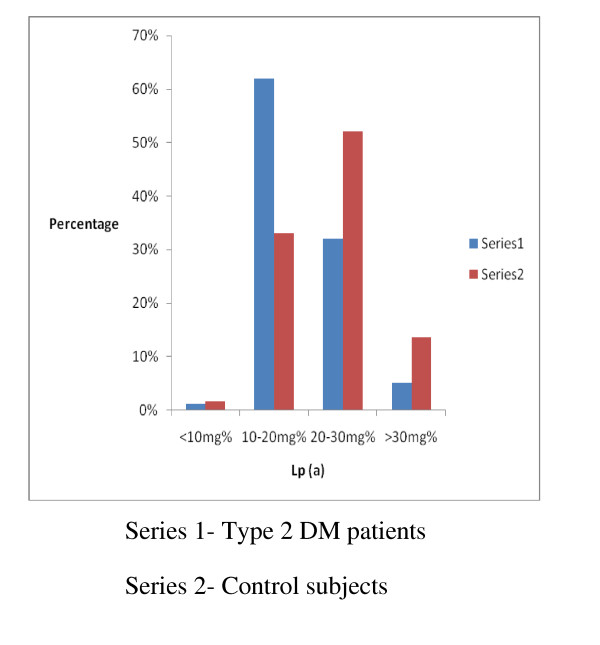
**Comparison of Lp (a) levels in type 2 DM subjects and Control subjects**.

**Figure 3:**
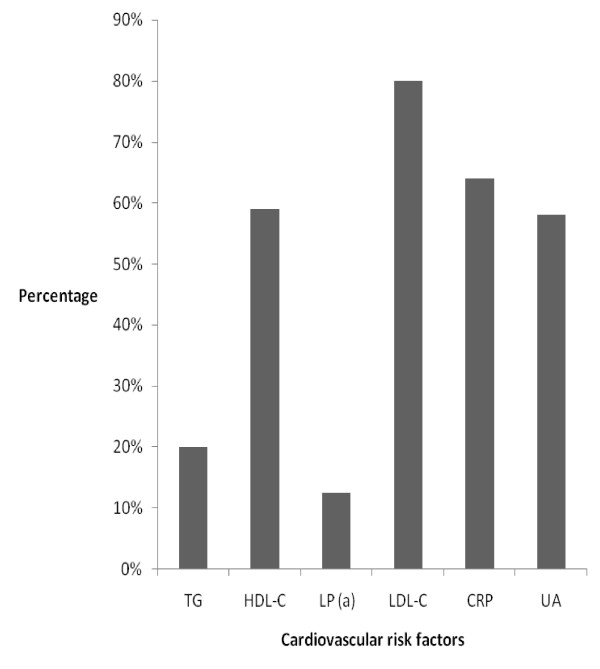
**Prevalence of cardiovascular risk factors in type 2 DM**.

The mean levels of Lp (a) in the subjects with type 2 DM were higher than those of the Control subjects and this difference was statistically significant (18.7 (5.8) mg/dl vs 23 (6.8) mg/dl, p-0.00001). The mean Lp (a ) levels in both sexes were however comparable (males vs females-22.9 (6.8) mg/dl vs 23.1 (6.7) mg/dl p-0.8. In subjects with and without hypertension the mean Lp (a) levels were also comparable (23 (6.7)mg/dl; vs 23 (6.8)mg/dl p-0.7). The prevalence of elevated Lp (a) levels in the subjects with type 2 DM was 12.5% and in the Control subjects was 4% and this difference was statistically significant, p-0.019. The majority of the study subjects had Lp(a) levels in the range of 20-30 mg/dl and a summary of these results are shown in Figure 1. A comparison of the distribution of the various categories of the Lp (a) levels showed that the proportion of the subjects with type 2 DM who had levels of Lp (a) greater than 20 mg/dl was greater than that of the Control subjects. (These results are shown in Figure 2). All studied biochemical parameters other than HDL-C were significantly higher in subjects with type 2 DM compared to the Control subjects. These results are shown in Table 2. A comparison of the mean levels of the biochemical parameters studied between type 2 DM subjects with elevated Lp (a) and those with normal Lp (a) levels showed that these two groups of subjects differed only in their TG levels. These results are shown in Table 3. Correlation analysis of Lp (a) and various parameters showed that Lp (a) was significantly correlated with some lipid parameters but had no significant association with glycosylated haemoglobin. These results are shown in Table 4. The pattern of distribution of CVS risk factors in the subjects with type 2 DM showed that uric acid and CRP were documented in 59% and 63% respectively of them. These results are depicted in Figure 3.

**Table 2:** Comparison of some biochemical parameters in subjects with type 2 DM and the Control subjects

Variable	Control subjects	Type 2 DM	p
HbA1c(%)	5(1.0)	6(2.3)	0.00001

TChol (mg%)	190.2(71.7)	314(52.8)	0.009

TG (mg%)	144.72(71.7)	300 (50.8)	0.007

HDL-C(mg%)	48.4(10)	41.8(14.7)	0.001

LDL-C(mg%)	123.5(20)	132(41.3)	0.04

LDL-C/HDL-C	2.6(0.7)	3.6 (1.8)	0.00001

CRP(mg%)	3.5(21)	9.2(13)	0.00001

SUA (mg/dL)	7.2(2.2)	7.2(2.8)	0.8

**Table 3:** Comparison of some clinical and biochemical parameters in subjects with normal and elevated Lp (a) levels

Parameter	Subjects with elevated Lp(a) levels	Subjects with normal elevated Lp(a levels)	p
HbA1c(%)	5.8(1.8)	6.4(2.4)	0.2

TChol (mg%)	209.5 (49)	196.4 (41.8)	0.1

TG (mg%)	144.72(71.7)	101 (52.8)	0.009

HDL-C(mg%)	38(14)	42.4(14.7)	0.1

LDL-C(mg%)	142.4(45.9)	130(40.6)	0.1

FBS(mg%)	166(79)	169(69.2)	0.8

CRP(mg% )	15.1 (24.1)	13.4(10.8)	0.9

SUA (mg/dL)	7.2(2.2)	7.2(2.8)	0.8

WC(cm)	95.8(13.7)	92.7(12.1)	0.2

BMI(Kg/m2)	29.3(7.3)	28.5(5.6)	0.5

DDM(years)	5.9(4.9)	6.1(6.1)	0.9

Age(years)	59.9(11.4)	57.2(10.3)	0.3

**Table 4:** Correlation (r) between LP (a) and some clinical and biochemical parameters

Variable	r	p
TG	0.2	0.007

HDL-C	-0.1	0.1

LDL-C	0.14	0.03

LDL-C/HDL-C	0.15	0.02

TCHOL	0.2	0.01

SUA	0.14	0.04

CRP	0.2	0.7

HbA1c	-0.01	0.5

## Discussion

We report the overall prevalence rate of elevated Lp (a) in the study subjects, patients with type 2 DM and the Control subjects to be 10.5%, 12.5% and 4% respectively. Unlike our findings, those by Scerthaner et al [18], noted that Lp (a) levels were comparable in subjects with DM and healthy Controls. Their findings may be attributed to the non-homogeneous nature of their study population which essentially was made up of subjects with types 1 and 2 DM. Many reports on Lp (a) levels however indicate higher prevalence rates of elevated Lp (a) in people with type 2 DM compared to healthy non diabetic subjects and type 1 DM patients [5,6,19]. Our reported prevalence rate of elevated Lp (a) is much lower than that by Habib et al [6] who reported a prevalence rate of 43.4% in people with type 2 DM. It is of note that elevated serum TG is a prominent feature of patients with type 2 DM who have elevated Lp (a) levels and this is evident by the results we obtained when we compared biochemical and clinical parameters between type 2 DM patients with elevated LP (a) levels and those with normal Lp (a) levels. Some studies however have shown that CRP is not only a significant correlate of Lp (a) but also suggest that it might be of clinical value in identifying individuals whose serum Lp(a) levels are transiently or chronically increased [20]. We have noted in this report that serum LP (a) had no significant correlation with CRP, but is significantly associated with other CVS risk factors namely, LDL-C, LDL/HDL, uric acid and TG. The findings by Heller et al[5] and Habib [6] differ somewhat from ours in that they report a positive correlation of Lp(a) with total cholesterol and LDL-C but not with triglycerides and HDL-C. We have also shown in this study that over half of our patients with type 2 DM have elevated CRP, LDL-C, SUA and reduced HDL-C levels. It is instructive to note that except for SUA, the mean serum levels of all the studied biochemical parameters (TCHOL, TG, HDL, LDL-C and LDL/HDL, CRP) differed significantly between the Control subjects and the subjects with type 2 DM. Hypertension a CVS risk factor and metabolic syndrome defining criterion is a commonly documented co-morbidity of DM in Nigerians [21,22]. We have found in this study, that the presence of hypertension did not affect Lp (a ) levels in DM subjects with and without hypertension. In an earlier Nigerian study [19], subjects with hypertensive-diabetes mellitus had significantly worse lipid and lipoprotein (a) profiles compared with subjects with diabetes mellitus or hypertension only. Although not stated as part of the objective of this study, a cursory assessment of our results showed that the presence of elevated Lp (a) levels had no bearing with glycaemic control and the mean HbA1c levels were comparable in DM patients with and without elevated Lp (a) levels. Some studies have [19,20,23] have shown similar results with our findings on the relationship between glycaemic control and Lp (a). In these studies [19,20,23] there was found no relation between HbA1c and Lp(a) concentrations in subjects with type 1 and 2 DM..

## Conclusion

Elevated serum Lp (a) levels are higher in subjects with DM than in people without DM but comparable in DM patients with and without hypertension. Serum LP (a) is significantly and positively associated with most of the atherogenic profile defining parameters in type 2 DM of which elevated TG is prominent.

## Competing interests

We, the authors declare that there are no competing interests.

## Authors' contributions

AOO designed the study, participated in data collation, statistical analysis, funding and writing the draft of the manuscript. AE participated in the laboratory analysis, funding, and data collation. All authors read and approved the final manuscript.
